# Supplementary figures and images for: The mechanism analysis of exogenous melatonin in limiting pear fruit aroma decrease under low temperature storage
Source: PeerJ. 2022 Oct 14;10:e14166. doi: 10.7717/peerj.14166 (PMC9575684; doi:10.7717/peerj.14166)

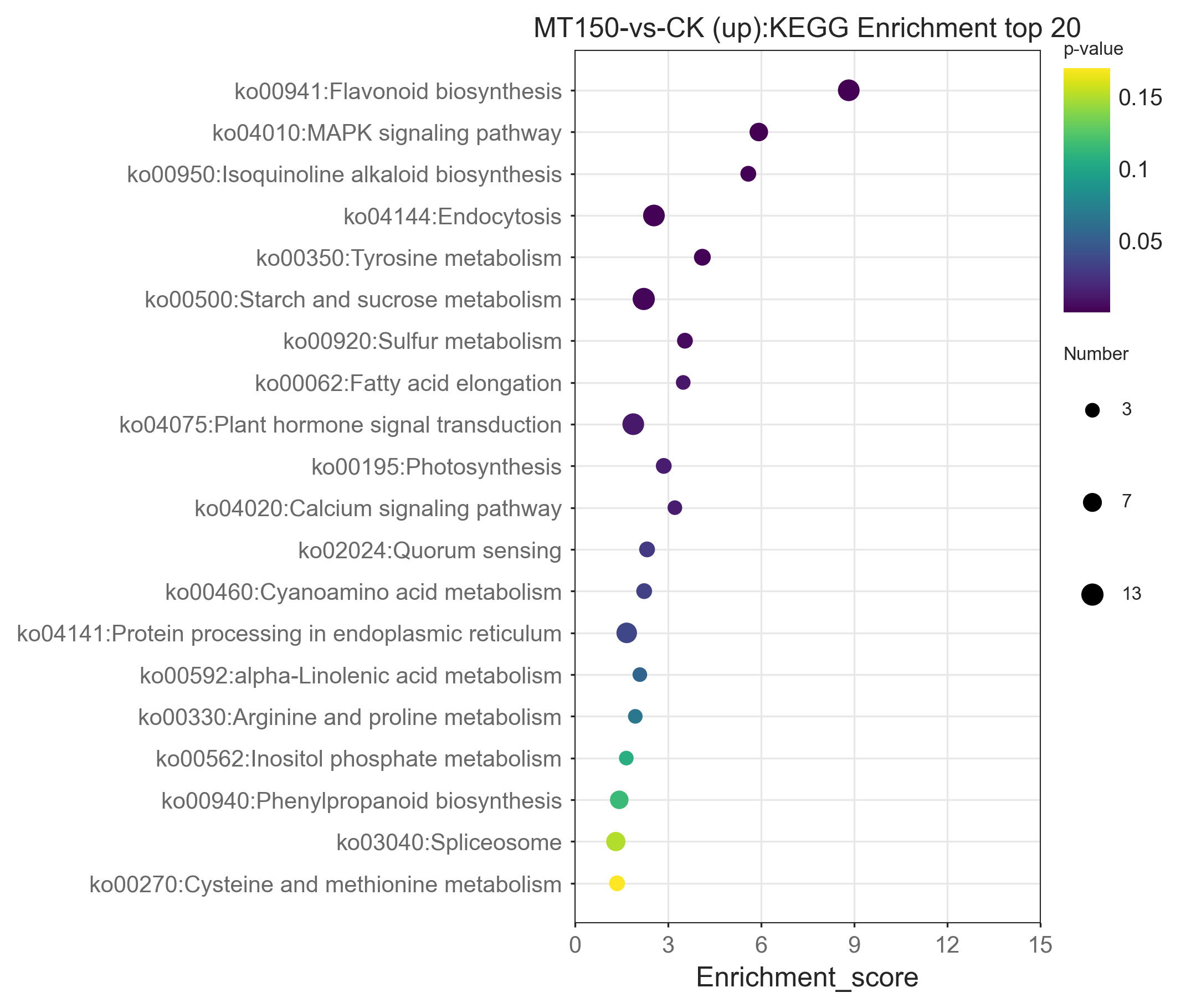

Supplement: Supplemental Information 1 [file peerj-10-14166-s001.png]

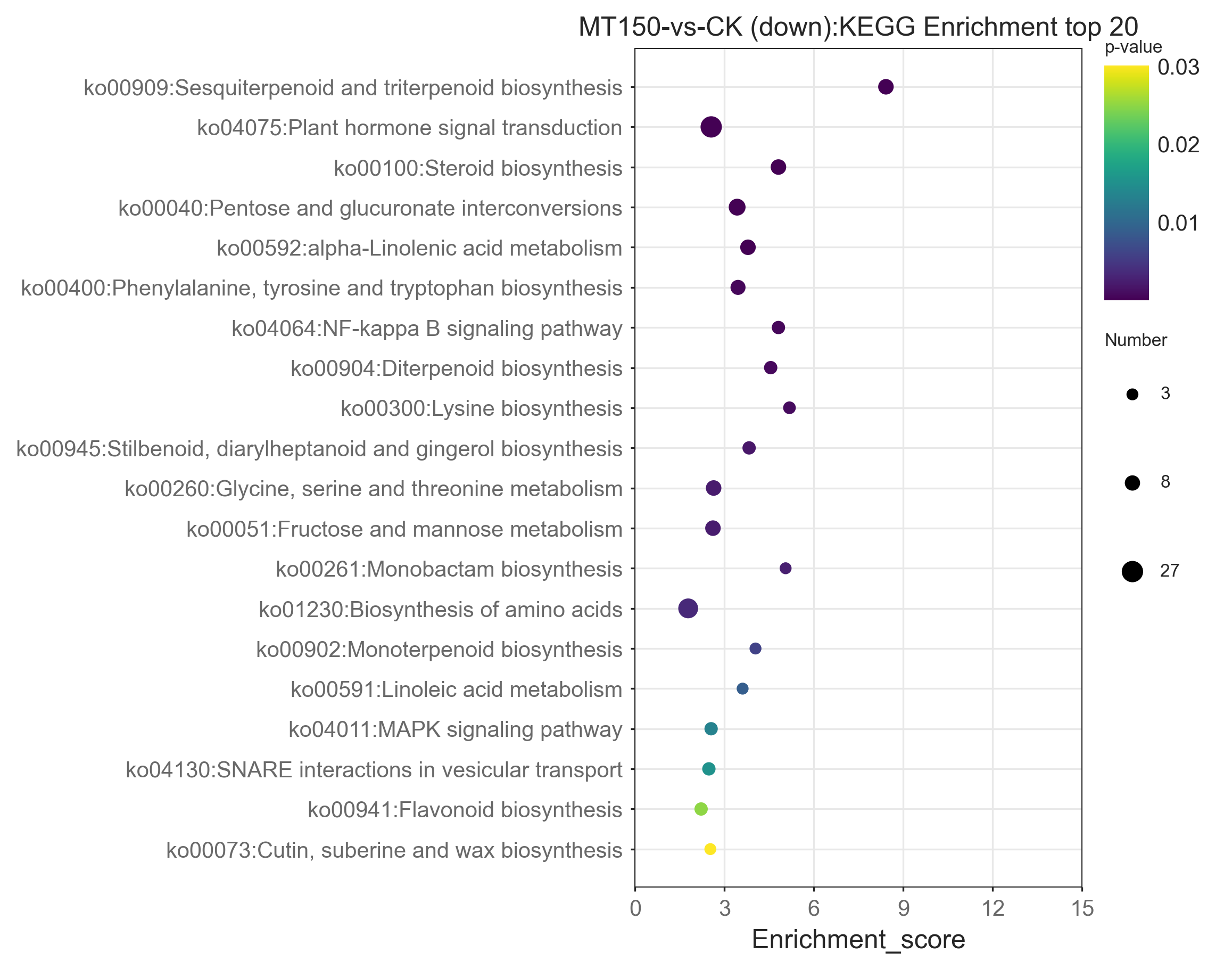

Supplement: Supplemental Information 2 [file peerj-10-14166-s002.png]
