# Supplementary material for: The mechanism analysis of exogenous melatonin in limiting pear fruit aroma decrease under low temperature storage
Source: PeerJ. 2022 Oct 14;10:e14166. doi: 10.7717/peerj.14166 (PMC9575684; doi:10.7717/peerj.14166)
Supplement: Supplemental Information 3 [file peerj-10-14166-s003.docx]

| **Table S1 The primers sequences used in this study** | | |  |
| --- | --- | --- | --- |
| **Gene ID** | **Forward primer（5‘-3’）** | **Reverse primer（5‘-3’）** | **Product length(bp)** |
| **pycom09g05270** | GTGGGATGTCGTTGTCTTC | AATCACCAGATAAAGGAGCAC | 92 |
| **pycom09g09820** | CCTGTGAAGTTCAAGGTGAGA | ACCAATTTGGTCAGCGAG | 108 |
| **pycom15g00250** | CCAATTCGGTATTCTACAGCAA | GGGCCTGAGAGGAAATGA | 90 |
| **pycom08g03050** | TCTGTGGTCTACGAAGCG | GAACCCGAACAGCAACTC | 132 |
| **pycom09g02160** | GAACAGCTGGTTGGCTTC | TTCCTAGTGGTACTGTTGCTAT | 81 |
| **pycom111g05530** | AAATGGACCAATAGGGCTG | GTATATGTCTAGCCGGGCG | 90 |
| **pycom17g24810** | TCCATTCTCATTCTCAAACACG | TGTGAGATGTTGGTGGTAGG | 97 |
| **pycom17g24820** | ATACCTTGTGAGCTCCGT | TTGCTTGTCGCCTCTCTAAT | 96 |
| **pycom07g24090** | CAAGACCATGAAATGTGCG | ACCAAATGCTTCACCTCG | 105 |
| **pycom06g10250** | TTGTAGCAAAGCATCCATCTC | GAAGCTGGGAAATTGTACGG | 80 |
