# Supplementary material for: The mechanism analysis of exogenous melatonin in limiting pear fruit aroma decrease under low temperature storage
Source: PeerJ. 2022 Oct 14;10:e14166. doi: 10.7717/peerj.14166 (PMC9575684; doi:10.7717/peerj.14166)
Supplement: Supplemental Information 4 [file peerj-10-14166-s004.docx]

**Table S2 Aroma substance of ‘Xinli No.7’ treated with different concentration of MT and stored at 4℃ for 80days**

| **Volatiles Name** | **Aroma content（ng·g^-1^）** | | | | | |
| --- | --- | --- | --- | --- | --- | --- |
|  | **CK** | **50μmol·L^-1^** | **100μmol·L^-1^** | **150 μmol·L^-1^** | **200μmol·L^-1^** | **250μmol·L^-1^** |
| **Esters** |  |  |  |  |  |  |
| 2,2,4-Trimethyl-1,3-pentanediol diisobutyrate | 0.144 | 0.112 | 0 | 0.089 | 0.154 | 0.118 |
| Acetic acid, butyl ester | 6.77 | 0 | 0.122 | 0.325 | 11.266 | 12.388 |
| Acetic acid, hexyl ester | 3.967 | 4.154 | 3.618 | 6.125 | 6.203 | 5.277 |
| Acetic acid, pentyl ester | 0.736 | 0 | 0 | 0.535 | 1.266 | 1.058 |
| n-Propyl acetate | 0.107 | 0 | 0.006 | 0.009 | 0.226 | 0.56 |
| Octadecanoic acid, | 0 | 0 | 0.855 | 0 | 0.237 | 0 |
| Ethyl Acetate | 0 | 0 | 0 | 0 | 0.655 | 0.626 |
| Isobutyl acetate | 0 | 0 | 0 | 0 | 0.356 | 0.263 |
| 1-Butanol, 3-methyl-, acetate | 0 | 0 | 0 | 0 | 0 | 4.325 |
| Isopropyl palmitate | 0 | 0 | 0 | 0 | 0.004 | 0 |
| **Esters No.** | 5 | 2 | 4 | 5 | 9 | 8 |
| **Ester’s content** | 11.724 | 4.266 | 4.601 | 7.083 | 20.367 | 24.615 |
| **Alcohols** |  |  |  |  |  |  |
| 1-Hexanol | 12.722 | 4.939 | 3.759 | 10.629 | 15.84 | 6.125 |
| 1-Octyn-3-ol, 4-ethyl- | 2.913 | 0 | 0 | 3.071 | 0 | 0 |
| 2-Octanol | 9.864 | 9.864 | 9.864 | 9.864 | 9.864 | 9.864 |
| 2-Propanol, 1-methoxy- | 0.001 | 0 | 0 | 0 | 0.007 | 0.011 |
| trans-2-Dodecen-1-ol | 0.035 | 0 | 0.043 | 0 | 0 | 0 |
| 1-Hexanol, 2-ethyl- | 0 | 3.271 | 2.718 | 0 | 2.712 | 2.743 |
| 1-Heptanol | 0 | 0 | 0 | 0.021 | 0 | 0.292 |
| 1-Hexanol, 5-methyl-2-(1-methylethyl)- | 0 | 0 | 0 | 0.007 | 0.109 | 0 |
| Sorbitol | 0 | 0 | 0 | 0.089 | 0.031 | 0 |
| 4-Heptanol, 3,4-dimethyl- | 0 | 0 | 0 | 0 | 0.772 | 0.785 |
| **Alcohols No.** | 5 | 3 | 4 | 6 | 7 | 6 |
| **Alcohols content** | 25.535 | 18.074 | 16.384 | 23.681 | 29.335 | 19.82 |
| **Aldehydes** |  |  |  |  |  |  |
| 13-Methyltetradecanal | 0.058 | 0 | 0 | 0.043 | 0 | 0 |
| 2-Hexenal, (E)- | 11.51 | 0.894 | 1.961 | 1.963 | 24.347 | 16.299 |
| Butanal, 2-methyl- | 0.375 | 0.713 | 0.897 | 0.615 | 0.472 | 0 |
| Butanal, 3-methyl- | 0.046 | 0 | 0.125 | 0.083 | 0.026 | 0.027 |
| Heptanal | 0.824 | 0.384 | 0.201 | 0.551 | 1.189 | 0.903 |
| Hexanal | 63.004 | 17.667 | 51.444 | 34.629 | 86.951 | 61.251 |
| Nonanal | 0.343 | 0.681 | 0.17 | 0.597 | 0.514 | 0.254 |
| Decanal | 0 | 0.664 | 0.23 | 1.165 | 0.571 | 0 |
| **Aldehydes No.** | 7 | 6 | 7 | 8 | 7 | 5 |
| **Aldehydes content** | 76.16 | 21.003 | 55.028 | 39.646 | 114.07 | 78.734 |
| **Ketones** |  |  |  |  |  |  |
| 2-Cyclohexen-1-one | 0.003 | 0 | 0.001 | 0 | 0 | 0 |
| 2-Octanone | 0.315 | 0.357 | 0.288 | 0.395 | 0.553 | 0.477 |
| 6-Hydroxyhexahydrocyclopenta[b]furan-2-one | 0.298 | 0 | 0.154 | 0 | 0 | 0 |
| Neocurdione | 0 | 0.002 | 0 | 0 | 0 | 0.002 |
| 2,3-Octanedione | 0 | 0 | 0 | 0.023 | 0.113 | 0 |
| **Ketones No.** | 3 | 2 | 3 | 2 | 2 | 2 |
| **Ketones content** | 0.616 | 0.359 | 0.443 | 0.418 | 0.666 | 0.479 |
| **Others** |  |  |  |  |  |  |
| 1,2,4-Methenoazulene | 0.053 | 0.044 | 0.023 | 0.016 | 0.017 | 0.032 |
| 1,2-Benzenediol, 3,5-bis(1,1-dimethylethyl)- | 0.018 | 0 | 0 | 0 | 0 | 0.297 |
| 2H-3,9a-Methano-1-benzoxepin | 0.052 | 0.004 | 0 | 0 | 0 | 0 |
| 8-Methyl-6-nonenoic acid | 0.015 | 0 | 0 | 0 | 0.504 | 0 |
| Cyclotrisiloxane, hexamethyl- | 10.513 | 0 | 0 | 0 | 0 | 0 |
| Dodecane | 0.124 | 0.108 | 0.078 | 0 | 0.109 | 0 |
| Dodecane, 2,6,11-trimethyl- | 0.022 | 0 | 0 | 0 | 0.019 | 0 |
| Ethylbenzene | 4.125 | 1.04 | 1.151 | 0.548 | 0 | 0 |
| Longifolene | 0.162 | 0.2 | 0.067 | 0 | 0 | 0 |
| n-Hexane | 0.195 | 0.161 | 0.21 | 0.24 | 0.189 | 0.171 |
| o-Xylene | 2.198 | 0.955 | 0.417 | 1.246 | 2.289 | 2.171 |
| Toluene | 1.439 | 0.573 | 0.788 | 0.847 | 1.077 | 0.55 |
| Undecane, 5-cyclohexyl- | 0.184 | 0.079 | 0 | 0 | 0 | 0 |
| Ylangene | 0.153 | 0 | 0 | 0 | 0.213 | 0.074 |
| Bicyclo[2.2.1]heptan-2-one, 1,7,7-trimethyl-, (1S)- | 0 | 0.057 | 0 | 0.024 | 0.055 | 0.04 |
| Butylated Hydroxytoluene | 0 | 0.133 | 0 | 0 | 0 | 0.076 |
| Fluorene | 0 | 0.203 | 0.064 | 0.271 | 0 | 0 |
| Methoprene | 0 | 0.019 | 0.011 | 0 | 0 | 0 |
| Hexadecane | 0 | 0 | 0.016 | 0 | 0.075 | 0 |
| Nonane, 5-(1-methylpropyl)- | 0 | 0 | 0.016 | 0.116 | 0 | 0 |
| Acenaphthene | 0 | 0 | 0 | 0.193 | 0.186 | 0.167 |
| Biphenylene | 0 | 0 | 0 | 0.192 | 0.178 | 0.255 |
| trans-beta-Ocimene | 0 | 0 | 0 | 0.164 | 0 | 0 |
| Nonane, 5-(2-methylpropyl)- | 0 | 0 | 0 | 0 | 0.259 | 0.404 |
| **Others No.** | 14 | 13 | 11 | 11 | 13 | 11 |
| **Others content** | 19.253 | 3.576 | 2.841 | 3.857 | 5.17 | 4.237 |
| **Total No.** | 34 | 26 | 29 | 32 | 38 | 32 |
| **Total content** | 133.288 | 47.278 | 79.297 | 74.685 | 169.608 | 127.885 |
