# Supplementary material for: The mechanism analysis of exogenous melatonin in limiting pear fruit aroma decrease under low temperature storage
Source: PeerJ. 2022 Oct 14;10:e14166. doi: 10.7717/peerj.14166 (PMC9575684; doi:10.7717/peerj.14166)
Supplement: Supplemental Information 5 [file peerj-10-14166-s005.docx]

**Table S3 List of comparisons between reads and reference genome**

|  | **CK1** | **CK2** | **CK3** | **MT1** | **MT2** | **MT3** |
| --- | --- | --- | --- | --- | --- | --- |
| Total reads | 51756450 | 49199146 | 50870742 | 56033446 | 55302270 | 56660982 |
| Total mapped reads | 46540825(89.92%) | 44293600(90.03%) | 45746178(89.93%) | 50249101(89.68%) | 49617446(89.72%) | 50606040(89.31%) |
| Multiple mapped | 1307667(2.53%) | 1187454(2.41%) | 1204882(2.37%) | 1403947(2.51%) | 1442869(2.61%) | 1430268(2.52%) |
| Uniquely mapped | 45233158(87.40%) | 43106146(87.62%) | 44541296(87.56%) | 48845154(87.17%) | 48174577(87.11%) | 49175772(86.79%) |
| Read-1 | 22586086(43.64%) | 21524869(43.75%) | 22239900(43.72%) | 24399563(43.54%) | 24058761(43.50%) | 24554445(43.34%) |
| Read-2 | 22647072(43.76%) | 21581277(43.87%) | 22301396(43.84%) | 24445591(43.63%) | 24115816(43.61%) | 24621327(43.45%) |
| Reads map to '+' | 22624540(43.71%) | 21562596(43.83%) | 22277399(43.79%) | 24428703(43.60%) | 24094158(43.57%) | 24589300(43.40%) |
| Reads map to '-' | 22608618(43.68%) | 21543550(43.79%) | 22263897(43.77%) | 24416451(43.57%) | 24080419(43.54%) | 24586472(43.39%) |
| Non-splice reads | 28075258(54.24%) | 26730094(54.33%) | 27596236(54.25%) | 29277133(52.25%) | 28866155(52.20%) | 29508514(52.08%) |
| Splice reads | 17157900(33.15%) | 16376052(33.29%) | 16945060(33.31%) | 19568021(34.92%) | 19308422(34.91%) | 19667258(34.71%) |
| Reads mapped in proper pairs | 42912762(82.91%) | 40920716(83.17%) | 42303914(83.16%) | 46359854(82.74%) | 45672544(82.59%) | 46656168(82.34%) |
