# Supplementary material for: The mechanism analysis of exogenous melatonin in limiting pear fruit aroma decrease under low temperature storage
Source: PeerJ. 2022 Oct 14;10:e14166. doi: 10.7717/peerj.14166 (PMC9575684; doi:10.7717/peerj.14166)
Supplement: Supplemental Information 8 [file peerj-10-14166-s008.docx]

**Table S5 List of gene ID enrichment in GO functional classification**

| **ID** | **Term** | **Genes** |
| --- | --- | --- |
| GO:0006468 | protein phosphorylation | pycom02g03820; pycom17g06000; pycom10g25870; pycom06g19560; pycom06g13700; pycom15g05520; pycom14g18920; pycom13g08370; pycom11g25970; pycom17g14920; pycom15g13120; pycom09g17690; pycom15g09760; pycom13g06730; pycom17g25000; pycom05g17490; pycom02g21130; pycom10g09500; pycom06g09540; pycom11g07040; pycom15g32960; pycom05g03200; pycom03g16570; pycom04g12430; pycom05g03220; pycom02g12330; pycom02g12340; pycom17g25060; pycom16g00670; pycom07g25640; pycom16g05270; pycom11g23530; pycom05g17120; pycom12g11840; pycom09g07690; pycom12g11850; pycom06g03780; pycom07g22310; pycom03g00260; pycom01g17370; pycom11g01710; pycom09g02260; pycom16g26340; pycom10g26460; pycom17g06200; pycom01g22480; pycom03g08680; pycom15g06990; pycom15g35130; pycom05g20170; pycom05g30270; pycom05g30280; pycom06g11830; pycom05g30290; pycom15g16680; pycom16g06630; pycom04g12150; pycom10g23160; pycom01g22960; pycom07g09340; pycom04g12170; pycom04g03330; pycom07g21170; pycom07g19890; pycom13g03150; pycom16g03300; pycom16g18420; pycom17g23120; pycom16g18430; pycom02g21350; pycom16g03320; pycom02g21360; pycom02g12520; pycom02g19200; pycom01g17070; pycom02g21370; pycom05g12270; pycom12g14060; pycom111g03320; pycom15g17590; pycom17g22770; pycom07g16590; pycom03g05000; pycom16g03380; pycom10g28270; pycom04g14780; pycom15g00830; pycom03g13460; pycom06g08540; pycom01g24290; pycom10g12770; pycom15g03330; pycom14g17130; pycom15g03340; pycom03g18070; pycom09g00350; pycom05g13160; pycom17g21560; pycom13g10860; pycom17g03900; pycom04g20720; pycom04g21120; pycom05g14000; pycom17g06400; pycom04g21130; pycom05g02290; pycom10g03130; pycom05g17820; pycom05g19920; pycom10g20840; pycom08g18820; pycom15g19360; pycom11g01980; pycom15g08430; pycom13g03300; pycom11g01990; pycom05g30920; pycom09g16380; pycom17g22480; pycom12g17570; pycom04g09390; pycom15g36220; pycom05g20870; pycom05g17020; pycom15g18150; pycom07g05390; pycom07g10460; pycom10g17070; pycom16g01450; pycom03g06450; pycom10g25940; pycom07g20140; pycom05g10800; pycom17g12440; pycom13g08410; pycom06g17560; pycom03g13640; pycom13g08420; pycom111g05640; pycom10g26370; pycom03g06490; pycom01g16030; pycom02g03950; pycom15g16560; pycom02g14000; pycom02g26640; pycom01g06840; pycom08g00930; pycom02g03980; pycom10g08760; pycom05g27220; pycom16g01980; pycom17g24290; pycom11g21030; pycom03g21710; pycom02g07320; pycom03g18740; pycom15g26710; pycom06g09650; pycom04g12530; pycom04g03280; pycom11g10140; pycom16g00750; pycom17g25150; pycom07g14380; pycom05g27710; pycom15g16220; pycom12g17790; pycom10g02120; pycom07g01390; pycom16g08300; pycom10g02140; pycom05g30300; pycom05g30310; pycom15g30600; pycom15g30610; pycom05g29000; pycom03g16310; pycom11g05590; pycom15g09560; pycom17g06310; pycom05g17720; pycom05g30380; pycom04g04660; pycom15g20140; pycom05g30390; pycom11g08550; pycom07g07310; pycom17g08440; pycom02g09150; pycom10g11910; pycom06g14450; pycom05g30840; pycom03g01710; pycom05g17780; pycom02g03330; pycom05g30860; pycom13g03250; pycom05g19890; pycom16g03410; pycom12g00710; pycom11g07350; pycom05g30890; pycom13g03280; pycom12g22590; pycom12g13750; pycom01g18860; pycom13g03290; pycom13g02010; pycom17g25350; pycom12g12070; pycom15g37890; pycom06g19550 |
| GO:0006355 | regulation of transcription, DNA-templated | pycom17g23290; pycom15g05090; pycom04g14890; pycom08g14230; pycom11g06160; pycom08g18430; pycom15g09730; pycom05g26710; pycom01g20680; pycom02g15660; pycom15g17340; pycom05g28850; pycom03g10280; pycom15g11080; pycom08g19310; pycom111g04390; pycom07g03330; pycom01g11010; pycom06g09580; pycom09g15200; pycom07g18480; pycom04g06130; pycom15g07300; pycom12g17690; pycom10g24300; pycom04g06140; pycom07g16830; pycom05g19210; pycom13g10630; pycom07g16840; pycom17g03260; pycom03g20460; pycom15g07350; pycom17g21370; pycom06g07110; pycom03g17920; pycom06g07120; pycom02g14950; pycom08g04760; pycom15g16650; pycom05g27300; pycom08g09390; pycom13g06920; pycom17g22700; pycom08g12390; pycom15g03690; pycom15g11250; pycom02g04920; pycom16g20580; pycom01g23800; pycom15g35660; pycom06g17320; pycom07g25810; pycom14g17960; pycom17g00490; pycom07g08150; pycom06g04350; pycom01g22600; pycom12g04870; pycom10g04320; pycom08g01980; pycom06g04360; pycom03g23120; pycom13g01590; pycom05g07690; pycom03g00470; pycom12g17060; pycom08g09510; pycom01g10030; pycom02g22700; pycom01g11730; pycom16g18100; pycom06g18260; pycom10g11550; pycom10g19920; pycom10g22900; pycom16g18120; pycom04g05140; pycom07g04490; pycom15g05950; pycom15g32890; pycom04g05190; pycom15g08450; pycom02g14340; pycom08g06230; pycom11g04930; pycom17g21220; pycom05g07360; pycom07g14630; pycom13g18490; pycom02g24040; pycom02g07650; pycom03g03950; pycom07g19240; pycom10g07840; pycom15g09350; pycom15g12330; pycom06g12970; pycom520g00050; pycom12g15980; pycom08g09230; pycom13g05940; pycom13g06340; pycom13g11410; pycom09g05110; pycom02g02260; pycom14g06400; pycom01g04730; pycom14g10690; pycom09g14410; pycom11g11370; pycom12g19370; pycom12g19370; pycom15g26280; pycom13g21150; pycom02g06940; pycom12g17730; pycom15g24630; pycom06g17220; pycom06g02110; pycom08g07690; pycom08g06410; pycom15g19580; pycom49g00070; pycom14g04850; pycom15g25080; pycom14g01500; pycom07g03490; pycom12g24110; pycom07g27020; pycom03g04580; pycom15g03700; pycom16g04170; pycom11g22430; pycom04g19770; pycom01g11670; pycom12g22080; pycom11g14010; pycom05g19830; pycom05g04720; pycom15g32790; pycom07g15770; pycom05g24920; pycom06g18640; pycom07g03150; pycom04g13960; pycom15g35720; pycom111g00470; pycom12g22560; pycom08g15420; pycom12g02810; pycom07g25900; pycom17g21150; pycom13g02000; pycom12g02820; pycom15g31590; pycom07g24220; pycom06g09890; pycom05g05640; pycom111g05530; pycom05g31740 |
| GO:0006952 | defense response | pycom16g02200; pycom16g13650; pycom15g20380; pycom07g19800; pycom13g13650; pycom04g09600; pycom16g00860; pycom16g00880; pycom04g04230; pycom04g04240; pycom290g00060; pycom11g10950; pycom08g13110; pycom04g09590; pycom13g13700; pycom13g13710; pycom01g13760; pycom13g13720; pycom13g13730; pycom16g13470; pycom13g13750; pycom12g12000; pycom01g16720; pycom04g09710; pycom13g13790; pycom10g24100 |
| GO:0016567 | protein ubiquitination | pycom17g26260; pycom13g17650; pycom13g10100; pycom05g01190; pycom16g07800; pycom07g22310; pycom01g03990; pycom12g03490; pycom13g11540; pycom05g27330; pycom15g02950; pycom08g06590; pycom14g20140; pycom10g22910; pycom15g06330; pycom01g13000; pycom14g03410; pycom111g03090; pycom14g03420; pycom05g15400; pycom10g13370; pycom07g16430; pycom1353g00040; pycom06g16000; pycom11g14800; pycom13g01490; pycom12g17420; pycom09g17950; pycom06g20780; pycom14g15480; pycom10g01460; pycom04g15230 |
| GO:0007165 | signal transduction | pycom01g20180; pycom05g13220; pycom12g00780; pycom10g02810; pycom111g02260; pycom07g21420; pycom15g16120; pycom02g25830; pycom17g21340; pycom02g19520; pycom17g08330; pycom10g00470; pycom10g06790; pycom10g01360; pycom15g33580; pycom05g01340; pycom10g01390; pycom05g01360; pycom05g01390; pycom02g02090; pycom11g06090; pycom10g02280; pycom02g25700; pycom16g09370; pycom01g20250; pycom15g04760; pycom09g02160; pycom17g21290; pycom111g05650; pycom05g13320; pycom04g02370; pycom05g14210; pycom04g01170; pycom02g17880; pycom12g22830; pycom12g22860; pycom02g22140; pycom02g22630; pycom02g22680; pycom04g00940; pycom10g01410; pycom07g23360; pycom10g01420; pycom11g18270; pycom13g17100; pycom17g21140; pycom01g20170; pycom10g02300 |
| GO:0006629 | lipid metabolic process | pycom05g25860; pycom05g25880; pycom02g13520; pycom04g04380; pycom05g25890; pycom04g02700; pycom15g08080; pycom01g10190; pycom01g10190; pycom07g12680; pycom07g12680; pycom17g06730; pycom15g24750; pycom15g24760; pycom08g08650; pycom05g24510; pycom11g06090; pycom03g14330; pycom03g14370; pycom02g13940; pycom17g01090; pycom07g20170; pycom15g08110; pycom05g04510; pycom09g18600; pycom16g17930; pycom07g12700; pycom07g12700; pycom06g04250; pycom07g16950; pycom04g13980; pycom10g27480; pycom08g08700 |
| GO:0048544 | recognition of pollen | pycom10g25870; pycom05g03200; pycom03g16570; pycom05g03210; pycom04g12430; pycom07g25630; pycom09g02260; pycom05g20170; pycom05g30270; pycom05g30280; pycom05g30290; pycom15g16680; pycom10g03130; pycom05g19920; pycom16g01450; pycom10g25940; pycom02g03950; pycom04g12530; pycom05g30310; pycom05g30380; pycom05g30390; pycom17g08440 |
| GO:0016126 | sterol biosynthetic process | pycom05g10720; pycom05g10730; pycom05g10770; pycom07g02910; pycom07g02920; pycom07g02930; pycom07g02940; pycom10g09930 |
| GO:0042546 | cell wall biogenesis | pycom15g26640; pycom15g17210; pycom10g22480; pycom13g11740; pycom16g06000; pycom13g22350; pycom09g07220; pycom09g07230; pycom17g13320; pycom16g23630; pycom16g23640 |
| GO:0006073 | cellular glucan metabolic process | pycom15g26640; pycom15g17210; pycom10g22480; pycom13g11740; pycom13g22350; pycom09g07220; pycom09g07230; pycom17g13320; pycom16g23630; pycom16g23640 |
| GO:0005618 | cell wall | pycom15g26640; pycom15g17210; pycom10g22480; pycom13g11740; pycom13g22350; pycom09g07220; pycom09g07230; pycom17g13320; pycom16g23630; pycom16g23640 |
| GO:0005576 | extracellular region | pycom15g29670; pycom05g21020; pycom10g11500; pycom10g11500; pycom11g04450; pycom17g04830; pycom13g06300; pycom07g21090; pycom05g12690; pycom05g12690; pycom04g11810; pycom15g06250; pycom12g13760; pycom01g18000 |
| GO:0048046 | apoplast | pycom15g26640; pycom11g08180; pycom15g17210; pycom10g22480; pycom13g11740; pycom13g22350; pycom09g07220; pycom09g07230; pycom17g13320; pycom16g23630; pycom16g23640 |
| GO:0030286 | dynein complex | pycom03g17400; pycom11g20720; pycom02g02850; pycom04g12610 |
| GO:0031012 | extracellular matrix | pycom16g07350; pycom07g16090; pycom13g07410 |
| GO:0031225 | anchored component of membrane | pycom13g13600; pycom17g06430; pycom12433g00030 |
| GO:0016020 | membrane | pycom07g24250; pycom07g15410; pycom07g15420; pycom09g07980; pycom07g24270; pycom07g24280; pycom03g19860; pycom11g05770; pycom111g04310; pycom15g05570; pycom14g18970; pycom11g12960; pycom10g21310; pycom15g29990; pycom15g04340; pycom04g14520; pycom04g14520; pycom09g16470; pycom05g03230; pycom12g13490; pycom10g21390; pycom02g22020; pycom12g06760; pycom07g14740; pycom17g28020; pycom15g27520; pycom10g15080; pycom10g06260; pycom07g22350; pycom10g06280; pycom17g03740; pycom15g38960; pycom10g10120; pycom06g08880; pycom17g24360; pycom17g19710; pycom12g11090; pycom06g11880; pycom15g25970; pycom15g37720; pycom16g15960; pycom10g28220; pycom05g17310; pycom09g07010; pycom10g04360; pycom12g03640; pycom12g03640; pycom15g39080; pycom17g09770; pycom12g03650; pycom12g03650; pycom02g11390; pycom15g14760; pycom15g14760; pycom13g11730; pycom08g21390; pycom15g35370; pycom15g35380; pycom13g16350; pycom13g16370; pycom15g16040; pycom13g03350; pycom16g06000; pycom15g06820; pycom16g07700; pycom12g11760; pycom12g11780; pycom05g17070; pycom05g17070; pycom13g15160; pycom14g03470; pycom14g03470; pycom14g03480; pycom14g03480; pycom01g13550; pycom14g16920; pycom14g16920; pycom15g19040; pycom09g11460; pycom08g21500; pycom10g26840; pycom10g26840; pycom10g27250; pycom111g01950; pycom13g08930; pycom02g03160; pycom11g11830; pycom03g08240; pycom05g21420; pycom11g22780; pycom13g22010; pycom06g17250; pycom10g06300; pycom461g00310; pycom461g00310; pycom07g25760; pycom10g06330; pycom01g10790; pycom17g03360; pycom16g19250; pycom16g19250; pycom16g03740; pycom14g20040; pycom06g04750; pycom16g05030; pycom01g16700; pycom15g13420; pycom15g17620; pycom15g13440; pycom02g03340; pycom14g00820; pycom08g04090; pycom16g03430; pycom10g28330; pycom05g28300; pycom05g06030; pycom05g28310 |
| GO:0000148 | 1,3-beta-D-glucan synthase complex | pycom09g16470; pycom17g24360; pycom09g07010 |
| GO:0000145 | exocyst | pycom09g09710; pycom04g10770; pycom03g04520; pycom11g09770; pycom12g12890; pycom17g00560 |
| GO:0016021 | integral component of membrane | pycom05g10720; pycom05g10730; pycom10g17450; pycom15g39150; pycom05g10770; pycom05g26700; pycom03g03090; pycom12g13420; pycom07g02910; pycom07g02920; pycom111g02290; pycom01g10190; pycom10g17970; pycom07g02930; pycom07g02940; pycom15g20380; pycom17g02810; pycom09g12740; pycom15g31750; pycom12g06760; pycom10g19240; pycom10g19240; pycom461g00250; pycom07g12680; pycom01g14010; pycom10g06260; pycom07g22350; pycom10g06280; pycom07g04270; pycom17g03760; pycom07g15660; pycom08g21610; pycom10g24840; pycom10g08860; pycom10g20680; pycom04g09600; pycom15g25970; pycom15g37720; pycom16g20590; pycom16g15960; pycom09g11210; pycom09g11210; pycom04g05900; pycom13g06120; pycom17g09770; pycom14g19730; pycom02g11840; pycom13g16350; pycom13g16370; pycom290g00060; pycom10g09930; pycom15g08940; pycom17g12440; pycom16g21660; pycom13g15160; pycom01g18130; pycom02g15270; pycom05g20080; pycom02g15280; pycom11g10950; pycom11g17640; pycom11g17640; pycom04g03230; pycom06g10500; pycom11g17650; pycom11g17650; pycom15g20410; pycom16g17930; pycom13g14420; pycom05g26870; pycom05g26870; pycom04g20970; pycom07g12700; pycom13g19040; pycom04g09590; pycom10g06300; pycom05g18490; pycom05g18490; pycom17g21420; pycom10g06330; pycom15g02860; pycom10g16000; pycom10g16000; pycom15g23460; pycom10g20710; pycom13g04450; pycom05g05970; pycom06g16500; pycom13g04460; pycom01g13760; pycom13g28840; pycom13g04470; pycom05g30810; pycom06g18620; pycom08g04020; pycom12g12000; pycom04g09710; pycom10g12350; pycom06g18670; pycom10g24100; pycom08g09100; pycom05g06030 |
| GO:0004672 | protein kinase activity | pycom02g03820; pycom17g06000; pycom10g25870; pycom06g19560; pycom06g13700; pycom15g05520; pycom14g18920; pycom13g08370; pycom11g25970; pycom17g14920; pycom15g13120; pycom09g17690; pycom15g09760; pycom13g06730; pycom17g25000; pycom05g17490; pycom02g21130; pycom10g09500; pycom06g09540; pycom11g07040; pycom15g32960; pycom05g03200; pycom03g16570; pycom04g12430; pycom05g03220; pycom02g12330; pycom02g12340; pycom17g25060; pycom16g00670; pycom07g25640; pycom16g05270; pycom11g23530; pycom05g17120; pycom12g11840; pycom09g07690; pycom12g11850; pycom06g03780; pycom07g22310; pycom03g00260; pycom01g17370; pycom11g01710; pycom09g02260; pycom16g26340; pycom10g26460; pycom17g06200; pycom01g22480; pycom03g08680; pycom15g06990; pycom15g35130; pycom05g20170; pycom05g30270; pycom05g30280; pycom06g11830; pycom05g30290; pycom15g16680; pycom16g06630; pycom04g12150; pycom10g23160; pycom01g22960; pycom07g09340; pycom04g12170; pycom04g03330; pycom07g21170; pycom07g19890; pycom13g03150; pycom16g03300; pycom16g18420; pycom17g23120; pycom16g18430; pycom02g21350; pycom16g03320; pycom02g21360; pycom02g12520; pycom02g19200; pycom01g17070; pycom02g21370; pycom05g12270; pycom12g14060; pycom111g03320; pycom15g17590; pycom17g22770; pycom07g16590; pycom03g05000; pycom16g03380; pycom10g28270; pycom04g14780; pycom15g00830; pycom03g13460; pycom06g08540; pycom01g24290; pycom10g12770; pycom15g03330; pycom14g17130; pycom15g03340; pycom03g18070; pycom09g00350; pycom05g13160; pycom17g21560; pycom13g10860; pycom17g03900; pycom04g20720; pycom04g21120; pycom05g14000; pycom17g06400; pycom04g21130; pycom05g02290; pycom10g03130; pycom05g17820; pycom05g19920; pycom10g20840; pycom08g18820; pycom15g19360; pycom11g01980; pycom15g08430; pycom13g03300; pycom11g01990; pycom05g30920; pycom09g16380; pycom17g22480; pycom12g17570; pycom04g09390; pycom15g36220; pycom05g20870; pycom05g17020; pycom15g18150; pycom07g05390; pycom07g10460; pycom10g17070; pycom16g01450; pycom03g06450; pycom10g25940; pycom07g20140; pycom05g10800; pycom17g12440; pycom13g08410; pycom06g17560; pycom03g13640; pycom13g08420; pycom111g05640; pycom10g26370; pycom03g06490; pycom01g16030; pycom02g03950; pycom15g16560; pycom02g14000; pycom02g26640; pycom01g06840; pycom08g00930; pycom02g03980; pycom10g08760; pycom05g27220; pycom16g01980; pycom17g24290; pycom11g21030; pycom03g21710; pycom02g07320; pycom03g18740; pycom15g26710; pycom06g09650; pycom04g12530; pycom04g03280; pycom11g10140; pycom16g00750; pycom17g25150; pycom07g14380; pycom05g27710; pycom15g16220; pycom12g17790; pycom10g02120; pycom07g01390; pycom16g08300; pycom10g02140; pycom05g30300; pycom05g30310; pycom15g30600; pycom15g30610; pycom05g29000; pycom03g16310; pycom11g05590; pycom15g09560; pycom17g06310; pycom05g17720; pycom05g30380; pycom04g04660; pycom15g20140; pycom05g30390; pycom11g08550; pycom07g07310; pycom17g08440; pycom02g09150; pycom10g11910; pycom06g14450; pycom05g30840; pycom03g01710; pycom05g17780; pycom02g03330; pycom05g30860; pycom13g03250; pycom05g19890; pycom16g03410; pycom12g00710; pycom11g07350; pycom05g30890; pycom13g03280; pycom12g22590; pycom12g13750; pycom01g18860; pycom13g03290; pycom13g02010; pycom17g25350; pycom12g12070; pycom15g37890; pycom06g19550 |
| GO:0003700 | DNA-binding transcription factor activity | pycom17g23290; pycom15g05090; pycom04g14890; pycom08g14230; pycom08g18430; pycom15g09730; pycom05g26710; pycom01g20680; pycom02g15660; pycom15g17340; pycom15g11080; pycom01g11010; pycom06g09580; pycom06g18840; pycom09g15200; pycom04g06130; pycom15g07300; pycom12g17690; pycom11g16370; pycom10g24300; pycom04g06140; pycom13g10630; pycom17g03260; pycom15g02760; pycom03g20460; pycom15g07350; pycom15g07350; pycom06g07110; pycom03g17920; pycom06g07120; pycom08g04760; pycom15g16650; pycom05g27300; pycom13g06920; pycom17g22700; pycom08g12390; pycom15g03690; pycom15g11250; pycom02g04920; pycom16g20580; pycom15g35660; pycom07g25810; pycom07g08150; pycom06g04350; pycom01g22600; pycom12g04870; pycom10g04320; pycom08g01980; pycom06g04360; pycom03g23120; pycom13g01590; pycom05g07690; pycom03g00470; pycom17g23660; pycom12g17060; pycom08g09510; pycom01g10030; pycom16g18100; pycom16g18100; pycom06g18260; pycom10g22900; pycom16g18120; pycom04g05140; pycom07g04490; pycom15g05950; pycom15g32890; pycom04g05190; pycom02g14340; pycom08g06230; pycom11g04930; pycom05g07360; pycom07g14630; pycom13g18490; pycom02g07650; pycom03g03950; pycom03g03950; pycom10g07840; pycom15g09350; pycom06g12970; pycom12g15980; pycom13g05940; pycom13g11410; pycom14g06400; pycom01g04730; pycom14g10690; pycom09g14410; pycom15g26280; pycom13g21150; pycom04g09530; pycom02g06940; pycom12g17730; pycom15g24630; pycom08g07690; pycom08g06410; pycom15g19580; pycom15g25080; pycom14g01500; pycom12g24110; pycom03g04580; pycom15g03700; pycom16g04170; pycom11g22430; pycom04g19770; pycom12g22080; pycom15g17180; pycom15g32790; pycom05g24920; pycom06g18640; pycom06g18640; pycom04g13960; pycom02g12160; pycom15g35720; pycom12g22560; pycom08g15420; pycom07g25900; pycom15g31590; pycom07g24220; pycom06g09890; pycom05g31740 |
| GO:0005524 | ATP binding | pycom02g03820; pycom02g03820; pycom06g08620; pycom17g06000; pycom17g06000; pycom10g25870; pycom10g25870; pycom06g19560; pycom06g19560; pycom06g13700; pycom15g05520; pycom14g18920; pycom13g08370; pycom13g08370; pycom111g04310; pycom111g04310; pycom11g25970; pycom11g25970; pycom17g14920; pycom17g14920; pycom15g13120; pycom11g12960; pycom09g17690; pycom15g09760; pycom15g09760; pycom13g06730; pycom17g25000; pycom05g17490; pycom05g17490; pycom02g21130; pycom10g09500; pycom06g09540; pycom06g09540; pycom11g07040; pycom11g07040; pycom15g32960; pycom05g03200; pycom05g03200; pycom03g16570; pycom03g16570; pycom04g12430; pycom04g12430; pycom05g03220; pycom05g03220; pycom02g12330; pycom02g12340; pycom17g25060; pycom16g00670; pycom07g25640; pycom12g20650; pycom12g20650; pycom16g05270; pycom16g05270; pycom11g23530; pycom12g06760; pycom05g17120; pycom05g17120; pycom12g11840; pycom12g11840; pycom09g07690; pycom09g07690; pycom17g02850; pycom12g11850; pycom12g11850; pycom06g03780; pycom06g03780; pycom07g22310; pycom07g22310; pycom03g00260; pycom03g00260; pycom10g15080; pycom01g17370; pycom11g01710; pycom11g01710; pycom09g02260; pycom09g02260; pycom16g26340; pycom02g11160; pycom10g26460; pycom17g06200; pycom02g11170; pycom01g22480; pycom01g22480; pycom03g08680; pycom03g08680; pycom07g04270; pycom15g06990; pycom15g06990; pycom15g35130; pycom02g11180; pycom05g20170; pycom13g04350; pycom02g22530; pycom05g30270; pycom05g30270; pycom05g30280; pycom06g11830; pycom05g30290; pycom15g16680; pycom16g06630; pycom04g12150; pycom04g12150; pycom10g23160; pycom10g23160; pycom15g36000; pycom01g22960; pycom07g09340; pycom04g12170; pycom04g12170; pycom04g03330; pycom04g03330; pycom15g25970; pycom07g21170; pycom07g21170; pycom07g19890; pycom07g19890; pycom15g37720; pycom13g03150; pycom13g03150; pycom16g03300; pycom16g03300; pycom16g18420; pycom16g18420; pycom17g23120; pycom17g23120; pycom16g18430; pycom16g18430; pycom02g21350; pycom02g21350; pycom16g03320; pycom16g03320; pycom02g21360; pycom02g21360; pycom02g12520; pycom02g12520; pycom02g19200; pycom02g19200; pycom01g17070; pycom01g17070; pycom02g21370; pycom02g21370; pycom14g15860; pycom05g12270; pycom12g14060; pycom12g14060; pycom111g03320; pycom15g17590; pycom15g17590; pycom09g11210; pycom17g22770; pycom15g26860; pycom07g16590; pycom07g16590; pycom03g05000; pycom16g03380; pycom10g28270; pycom04g14780; pycom15g00830; pycom03g13460; pycom03g13460; pycom06g08540; pycom01g24290; pycom10g12770; pycom15g03330; pycom14g17130; pycom14g17130; pycom15g03340; pycom15g03340; pycom03g18070; pycom03g18070; pycom09g00350; pycom05g13160; pycom17g21560; pycom17g21560; pycom14g01640; pycom13g10860; pycom13g10860; pycom17g03900; pycom04g20720; pycom04g20720; pycom04g21120; pycom05g14000; pycom17g06400; pycom17g06400; pycom04g21130; pycom05g02290; pycom05g02290; pycom10g03130; pycom10g03130; pycom05g17820; pycom05g17820; pycom05g19920; pycom10g20840; pycom10g20840; pycom08g18820; pycom08g18820; pycom15g19360; pycom11g01980; pycom11g01980; pycom15g08430; pycom15g08430; pycom13g03300; pycom13g03300; pycom11g01990; pycom11g01990; pycom15g22350; pycom05g30920; pycom09g16380; pycom09g16380; pycom17g22480; pycom12g17570; pycom04g09390; pycom04g09390; pycom14g14310; pycom15g36220; pycom15g36220; pycom05g20870; pycom05g17020; pycom05g17020; pycom15g18150; pycom07g05390; pycom07g05390; pycom07g10460; pycom10g17070; pycom16g01450; pycom16g01450; pycom03g06450; pycom10g25940; pycom10g25940; pycom07g20140; pycom07g20140; pycom05g10800; pycom05g10800; pycom17g12440; pycom13g08410; pycom13g08410; pycom06g17560; pycom06g17560; pycom03g13640; pycom13g08420; pycom13g08420; pycom17g04010; pycom111g05640; pycom111g05640; pycom10g26370; pycom03g06490; pycom01g16030; pycom01g16030; pycom11g15070; pycom02g03950; pycom15g16560; pycom15g16560; pycom17g04040; pycom02g14000; pycom02g14000; pycom111g02300; pycom02g26640; pycom02g26640; pycom07g26900; pycom15g16580; pycom01g06840; pycom08g00930; pycom02g03980; pycom10g08760; pycom10g08760; pycom11g17640; pycom05g27220; pycom16g01980; pycom11g17650; pycom17g24290; pycom17g24290; pycom11g21030; pycom11g21030; pycom11g17660; pycom01g07280; pycom03g21710; pycom03g21710; pycom02g07320; pycom03g18740; pycom02g02750; pycom15g26710; pycom06g09650; pycom05g26870; pycom11g11830; pycom14g06910; pycom04g12530; pycom04g12530; pycom04g03280; pycom04g03280; pycom11g10140; pycom11g10140; pycom16g00750; pycom16g00750; pycom17g25150; pycom17g25150; pycom07g14380; pycom05g27710; pycom15g16220; pycom12g17790; pycom12g17790; pycom10g02120; pycom07g01390; pycom07g01390; pycom16g08300; pycom16g08300; pycom10g02140; pycom10g02140; pycom05g30300; pycom05g30310; pycom15g30600; pycom15g30600; pycom15g30610; pycom15g30610; pycom05g29000; pycom05g29000; pycom03g16310; pycom11g05590; pycom07g27020; pycom15g09560; pycom17g06310; pycom17g06310; pycom13g04450; pycom12397g00150; pycom12397g00150; pycom06g03060; pycom13g04460; pycom05g17720; pycom05g17720; pycom05g30380; pycom05g30380; pycom04g04660; pycom04g04660; pycom16g25200; pycom13g04470; pycom01g23420; pycom15g20140; pycom15g20140; pycom05g30390; pycom05g30390; pycom11g08550; pycom11g08550; pycom07g07310; pycom07g07310; pycom17g08440; pycom17g08440; pycom02g09150; pycom02g09150; pycom10g11910; pycom10g11910; pycom11g03100; pycom06g14450; pycom05g30840; pycom05g30840; pycom03g01710; pycom03g01710; pycom05g17780; pycom05g17780; pycom13g11690; pycom02g03330; pycom05g30860; pycom05g30860; pycom06g18670; pycom13g03250; pycom13g03250; pycom16g02590; pycom05g18190; pycom05g19890; pycom16g03410; pycom16g03410; pycom12g00710; pycom111g01300; pycom11g07350; pycom05g30890; pycom05g30890; pycom13g03280; pycom13g03280; pycom111g03400; pycom12g22590; pycom12g13750; pycom12g13750; pycom01g18860; pycom13g03290; pycom13g02010; pycom17g25350; pycom12g12070; pycom12g12070; pycom07g03650; pycom15g37890; pycom15g37890; pycom06g19550 |
| GO:0043565 | sequence-specific DNA binding | pycom17g23290; pycom15g05090; pycom04g14890; pycom15g09730; pycom05g26710; pycom04g14060; pycom02g15660; pycom01g11010; pycom09g15200; pycom03g09870; pycom15g07300; pycom12g17690; pycom07g16830; pycom13g10630; pycom17g03260; pycom03g20460; pycom08g04760; pycom15g16650; pycom05g27300; pycom08g09390; pycom13g06920; pycom17g22700; pycom15g03690; pycom16g20580; pycom01g22600; pycom12g04870; pycom13g01590; pycom12g17060; pycom01g10030; pycom10g22900; pycom15g05950; pycom15g32890; pycom02g14340; pycom08g06230; pycom11g04930; pycom07g14630; pycom06g12970; pycom12g10510; pycom12g15980; pycom13g05940; pycom09g05110; pycom14g06400; pycom14g10690; pycom09g14410; pycom12g19370; pycom13g21150; pycom12g17730; pycom06g02110; pycom08g07690; pycom12g16050; pycom15g25080; pycom03g04580; pycom15g03700; pycom05g04720; pycom05g24920; pycom04g13960; pycom07g25900; pycom06g09890; pycom05g31740 |
| GO:0043531 | ADP binding | pycom10g02310; pycom05g13220; pycom12g00780; pycom06g01970; pycom06g01980; pycom10g02340; pycom15g24030; pycom15g24050; pycom15g24070; pycom05g12010; pycom10g02810; pycom07g21420; pycom11g05400; pycom15g16120; pycom11g05420; pycom02g06010; pycom02g25830; pycom02g11110; pycom17g21340; pycom08g06820; pycom08g06850; pycom07g22380; pycom06g09270; pycom10g00470; pycom10g01360; pycom15g33580; pycom05g01340; pycom08g10780; pycom02g22200; pycom10g01390; pycom05g01360; pycom02g22230; pycom05g01390; pycom02g02090; pycom04g21110; pycom14g05860; pycom03g01390; pycom05g09430; pycom06g10800; pycom08g10910; pycom02g25710; pycom15g04720; pycom05g14530; pycom01g20250; pycom17g21290; pycom17g05700; pycom05g13320; pycom04g02350; pycom04g02370; pycom08g08030; pycom05g14210; pycom04g01170; pycom02g17880; pycom12g22830; pycom12g22860; pycom02g22140; pycom02g13300; pycom02g13310; pycom02g22630; pycom05g04280; pycom05g13540; pycom02g22680; pycom04g00930; pycom04g00940; pycom17g15640; pycom10g01410; pycom15g23980; pycom10g01420; pycom11g18270; pycom13g17100; pycom17g21140; pycom03g02170; pycom10g02300 |
| GO:0004842 | ubiquitin-protein transferase activity | pycom17g26260; pycom13g17650; pycom13g10100; pycom05g01190; pycom16g07800; pycom07g22310; pycom01g03990; pycom12g03490; pycom13g11540; pycom05g27330; pycom15g02950; pycom08g06590; pycom14g20140; pycom10g22910; pycom15g06330; pycom01g13000; pycom14g03410; pycom111g03090; pycom14g03420; pycom05g15400; pycom10g13370; pycom07g16430; pycom1353g00040; pycom06g16000; pycom11g14800; pycom13g01490; pycom12g17420; pycom09g17950; pycom06g20780; pycom14g15480; pycom10g01460; pycom04g15230 |
| GO:0050660 | flavin adenine dinucleotide binding | pycom05g10720; pycom05g10730; pycom10g20420; pycom10g20420; pycom10g20430; pycom10g20430; pycom09g10980; pycom05g10770; pycom05g25010; pycom05g25010; pycom05g25020; pycom05g25020; pycom10g20480; pycom10g20480; pycom05g25050; pycom05g25050; pycom07g02910; pycom07g02920; pycom07g02930; pycom07g02940; pycom11g08780; pycom15g04860; pycom15g04860; pycom15g16630; pycom14g17110; pycom10g09930; pycom15g09030; pycom05g13010; pycom08g01870; pycom08g01870; pycom10g15190; pycom16g00480; pycom16g00480 |
| GO:0004506 | squalene monooxygenase activity | pycom05g10720; pycom05g10730; pycom05g10770; pycom07g02910; pycom07g02920; pycom07g02930; pycom07g02940; pycom10g09930 |
| GO:0004674 | protein serine/threonine kinase activity | pycom10g25870; pycom03g16570; pycom15g35130; pycom05g20170; pycom05g30270; pycom05g30280; pycom15g16680; pycom10g20680; pycom17g21560; pycom05g19920; pycom11g01990; pycom16g01450; pycom10g25940; pycom17g12440; pycom05g30310; pycom10g20710; pycom05g30380; pycom05g30390; pycom17g08440; pycom03g01710; pycom13g03250; pycom05g19890 |
| GO:0003677 | DNA binding | pycom09g19720; pycom08g14230; pycom11g06160; pycom08g18430; pycom14g15170; pycom09g10130; pycom01g20680; pycom07g13820; pycom02g15660; pycom15g17340; pycom05g28850; pycom03g10280; pycom15g11080; pycom08g19310; pycom16g18240; pycom111g04390; pycom01g11010; pycom06g09580; pycom02g08980; pycom07g18480; pycom07g15110; pycom13g09720; pycom04g06130; pycom12g18500; pycom10g24300; pycom04g06140; pycom07g16830; pycom07g16840; pycom07g16840; pycom09g02230; pycom17g03260; pycom15g07350; pycom06g07110; pycom03g17920; pycom06g07120; pycom08g08930; pycom07g04700; pycom08g09360; pycom09g18240; pycom13g06480; pycom08g09390; pycom15g08260; pycom13g06920; pycom08g12390; pycom14g11620; pycom07g00580; pycom15g11250; pycom02g04920; pycom14g20470; pycom05g08850; pycom01g23800; pycom15g35660; pycom06g17320; pycom07g25810; pycom14g17960; pycom10g24050; pycom17g00490; pycom11g06890; pycom14g18380; pycom07g08150; pycom11g08120; pycom06g04350; pycom12g04870; pycom10g04320; pycom06g04360; pycom03g23120; pycom02g13400; pycom16g12660; pycom03g00470; pycom02g04180; pycom08g09510; pycom01g11730; pycom16g18100; pycom06g18260; pycom10g11550; pycom10g19920; pycom16g18120; pycom04g05140; pycom09g16340; pycom07g04490; pycom15g05950; pycom04g05190; pycom08g06230; pycom06g10800; pycom08g05850; pycom05g07360; pycom16g25380; pycom01g17250; pycom13g18490; pycom02g07650; pycom03g03950; pycom07g19240; pycom07g19240; pycom10g07840; pycom15g12330; pycom520g00050; pycom13g06320; pycom13g06340; pycom13g11410; pycom11g02050; pycom05g30170; pycom15g27900; pycom01g04730; pycom15g23740; pycom17g24260; pycom11g11370; pycom11g11370; pycom12g19370; pycom12g19370; pycom10g15950; pycom02g11590; pycom02g06940; pycom15g24630; pycom06g17220; pycom06g02110; pycom08g06410; pycom05g18450; pycom15g19580; pycom15g16210; pycom49g00070; pycom05g21440; pycom10g24400; pycom14g01500; pycom17g27710; pycom16g04130; pycom12g24110; pycom02g26380; pycom16g04170; pycom12g13210; pycom11g22430; pycom17g08410; pycom17g19350; pycom01g11670; pycom11g14010; pycom04g13920; pycom05g19830; pycom05g04720; pycom15g32790; pycom07g15770; pycom03g23560; pycom06g18640; pycom15g35720; pycom13g11690; pycom111g00470; pycom12g22560; pycom08g15420; pycom14g02910; pycom12g02810; pycom13g02000; pycom13g02000; pycom12g02820; pycom15g07140; pycom15g31590; pycom07g24220 |
