# Supplementary material for: The mechanism analysis of exogenous melatonin in limiting pear fruit aroma decrease under low temperature storage
Source: PeerJ. 2022 Oct 14;10:e14166. doi: 10.7717/peerj.14166 (PMC9575684; doi:10.7717/peerj.14166)
Supplement: Supplemental Information 9 [file peerj-10-14166-s009.docx]

**TableS6 List of important metabolic pathways of DEGs**

| **Pathway ID** | **Pathway Name** | **All genes with pathway annotation** | **DEGs with pathway annotation** |
| --- | --- | --- | --- |
|  |  |  |  |
|  |  |  |  |
| **Fatty acid metabolism** |  |  |  |
| ko00061 | Fatty acid biosynthesis | 72 | 2 |
| ko00062 | Fatty acid elongation | 48 | 6 |
| ko00071 | Fatty acid degradation | 43 | 3 |
| ko00540 | Lipopolysaccharide biosynthesis | 12 | ND |
| ko00561 | Glycerolipid metabolism | 106 | 4 |
| ko00565 | Ether lipid metabolism | 39 | 3 |
| ko00590 | Arachidonic acid metabolism | 23 | ND |
| ko00591 | Linoleic acid metabolism | 28 | 4 |
| ko00592 | alpha-Linolenic acid metabolism | 80 | 12 |
| ko00600 | Sphingolipid metabolism | 46 | ND |
| ko00785 | Lipoic acid metabolism | 9 | ND |
| ko00903 | Limonene and pinene degradation | 11 | 1 |
| ko01040 | Biosynthesis of unsaturated fatty acids | 56 | 1 |
| ko01212 | Fatty acid metabolism | 123 | 2 |
| **Transcription factor** |  |  |  |
| ko03022 | Basal transcription factors | 72 | 1 |
| **Amino acid metabolism** |  |  |  |
| ko00220 | Arginine biosynthesis | 51 | 3 |
| ko00250 | Alanine, aspartate and glutamate metabolism | 78 | 3 |
| ko00260 | Glycine, serine and threonine metabolism | 115 | 9 |
| ko00270 | Cysteine and methionine metabolism | 164 | 12 |
| ko00280 | Valine, leucine and isoleucine degradation | 77 | 2 |
| ko00290 | Valine, leucine and isoleucine biosynthesis | 34 | 1 |
| ko00300 | Lysine biosynthesis | 26 | 4 |
| ko00310 | Lysine degradation | 50 | 1 |
| ko00330 | Arginine and proline metabolism | 86 | 6 |
| ko00340 | Histidine metabolism | 35 | 1 |
| ko00350 | Tyrosine metabolism | 68 | 5 |
| ko00360 | Phenylalanine metabolism | 65 | 4 |
| ko00380 | Tryptophan metabolism | 55 | 3 |
| ko00400 | Phenylalanine, tyrosine and tryptophan biosynthesis | 78 | 10 |
| ko00410 | beta-Alanine metabolism | 73 | 2 |
| ko00430 | Taurine and hypotaurine metabolism | 18 | 2 |
| ko00471 | D-Glutamine and D-glutamate metabolism | 8 | 1 |
| ko00480 | Glutathione metabolism | 132 | 3 |
| ko00510 | N-Glycan biosynthesis | 74 | 2 |
| ko00620 | Pyruvate metabolism | 153 | 1 |
| ko00730 | Thiamine metabolism | 25 | ND |
| ko01230 | Biosynthesis of amino acids | 397 | 25 |
| **Carbohydrate metabolism** |  |  |  |
| ko00051 | Fructose and mannose metabolism | 116 | 11 |
| ko00052 | Galactose metabolism | 81 | 3 |
| ko00500 | Starch and sucrose metabolism | 327 | 26 |
| ko00511 | Other glycan degradation | 31 | ND |
| **Hormone and calcium signal** |  |  |  |
| ko00905 | Brassinosteroid biosynthesis | 29 | 2 |
| ko00908 | Zeatin biosynthesis | 17 | 4 |
| ko04020 | Calcium signaling pathway | 52 | 4 |
| ko04075 | Plant hormone signal transduction | 356 | 39 |
| **Flavonoids** |  |  |  |
| ko00941 | Flavonoid biosynthesis | 76 | 17 |
| ko00944 | Flavone and flavonol biosynthesis | 3 | 2 |
| **Others** |  |  |  |
| ko00010 | Glycolysis / Gluconeogenesis | 209 | 5 |
| ko00020 | Citrate cycle (TCA cycle) | 111 | ND |
| ko00030 | Pentose phosphate pathway | 82 | 5 |
| ko00040 | Pentose and glucuronate interconversions | 118 | 14 |
| ko00053 | Ascorbate and aldarate metabolism | 69 | 4 |
| ko00072 | Synthesis and degradation of ketone bodies | 8 | ND |
| ko00073 | Cutin, suberine and wax biosynthesis | 40 | 5 |
| ko00100 | Steroid biosynthesis | 63 | 9 |
| ko00130 | Ubiquinone and other terpenoid-quinone biosynthesis | 61 | 3 |
| ko00140 | Steroid hormone biosynthesis | 12 | 1 |
| ko00190 | Oxidative phosphorylation | 195 | 3 |
| ko00195 | Photosynthesis | 78 | 5 |
| ko00196 | Photosynthesis - antenna proteins | 28 | 1 |
| ko00230 | Purine metabolism | 259 | 4 |
| ko00240 | Pyrimidine metabolism | 183 | 4 |
| ko00261 | Monobactam biosynthesis | 20 | 3 |
| ko00281 | Geraniol degradation | 3 | ND |
| ko00401 | Novobiocin biosynthesis | 5 | ND |
| ko00440 | Phosphonate and phosphinate metabolism | 11 | 1 |
| ko00450 | Selenocompound metabolism | 33 | 1 |
| ko00460 | Cyanoamino acid metabolism | 100 | 4 |
| ko00513 | Various types of N-glycan biosynthesis | 54 | ND |
| ko00514 | Other types of O-glycan biosynthesis | 6 | 1 |
| ko00520 | Amino sugar and nucleotide sugar metabolism | 212 | 12 |
| ko00521 | Streptomycin biosynthesis | 22 | ND |
| ko00524 | Neomycin, kanamycin and gentamicin biosynthesis | 10 | ND |
| ko00531 | Glycosaminoglycan degradation | 17 | ND |
| ko00532 | Glycosaminoglycan biosynthesis - chondroitin sulfate / dermatan sulfate | 2 | 1 |
| ko00534 | Glycosaminoglycan biosynthesis - heparan sulfate / heparin | 2 | 1 |
| ko00562 | Inositol phosphate metabolism | 101 | 6 |
| ko00563 | Glycosylphosphatidylinositol (GPI)-anchor biosynthesis | 47 | 1 |
| ko00564 | Glycerophospholipid metabolism | 134 | 10 |
| ko00603 | Glycosphingolipid biosynthesis - globo series | 14 | 1 |
| ko00625 | Chloroalkane and chloroalkene degradation | 18 | 1 |
| ko00626 | Naphthalene degradation | 8 | ND |
| ko00630 | Glyoxylate and dicarboxylate metabolism | 112 | ND |
| ko00640 | Propanoate metabolism | 57 | 1 |
| ko00643 | Styrene degradation | 12 | ND |
| ko00650 | Butanoate metabolism | 28 | 1 |
| ko00670 | One carbon pool by folate | 34 | 1 |
| ko00680 | Methane metabolism | 94 | 4 |
| ko00710 | Carbon fixation in photosynthetic organisms | 115 | 4 |
| ko00720 | Carbon fixation pathways in prokaryotes | 62 | ND |
| ko00740 | Riboflavin metabolism | 11 | ND |
| ko00750 | Vitamin B6 metabolism | 20 | 2 |
| ko00760 | Nicotinate and nicotinamide metabolism | 27 | ND |
| ko00770 | Pantothenate and CoA biosynthesis | 48 | ND |
| ko00780 | Biotin metabolism | 32 | ND |
| ko00790 | Folate biosynthesis | 29 | 2 |
| ko00830 | Retinol metabolism | 20 | ND |
| ko00860 | Porphyrin and chlorophyll metabolism | 67 | 3 |
| ko00900 | Terpenoid backbone biosynthesis | 71 | 3 |
| ko00902 | Monoterpenoid biosynthesis | 25 | 5 |
| ko00904 | Diterpenoid biosynthesis | 37 | 6 |
| ko00906 | Carotenoid biosynthesis | 60 | 5 |
| ko00909 | Sesquiterpenoid and triterpenoid biosynthesis | 36 | 9 |
| ko00910 | Nitrogen metabolism | 55 | 5 |
| ko00920 | Sulfur metabolism | 63 | 5 |
| ko00940 | Phenylpropanoid biosynthesis | 273 | 20 |
| ko00945 | Stilbenoid, diarylheptanoid and gingerol biosynthesis | 44 | 6 |
| ko00950 | Isoquinoline alkaloid biosynthesis | 40 | 4 |
| ko00960 | Tropane, piperidine and pyridine alkaloid biosynthesis | 44 | 5 |
| ko00965 | Betalain biosynthesis | 3 | 1 |
| ko00966 | Glucosinolate biosynthesis | 4 | ND |
| ko00970 | Aminoacyl-tRNA biosynthesis | 157 | 1 |
| ko00980 | Metabolism of xenobiotics by cytochrome P450 | 65 | 3 |
| ko00982 | Drug metabolism - cytochrome P450 | 70 | 4 |
| ko00983 | Drug metabolism - other enzymes | 35 | 1 |
| ko01053 | Biosynthesis of siderophore group nonribosomal peptides | 2 | 1 |
| ko01200 | Carbon metabolism | 439 | 11 |
| ko01210 | 2-Oxocarboxylic acid metabolism | 95 | 5 |
| ko01220 | Degradation of aromatic compounds | 14 | 1 |
| ko02010 | ABC transporters | 41 | 1 |
| ko02020 | Two-component system | 19 | ND |
| ko02024 | Quorum sensing | 96 | 9 |
| ko03008 | Ribosome biogenesis in eukaryotes | 140 | ND |
| ko03010 | Ribosome | 400 | 3 |
| ko03013 | RNA transport | 278 | 2 |
| ko03015 | mRNA surveillance pathway | 204 | 2 |
| ko03018 | RNA degradation | 175 | 7 |
| ko03020 | RNA polymerase | 69 | ND |
| ko03030 | DNA replication | 104 | 2 |
| ko03040 | Spliceosome | 338 | 9 |
| ko03050 | Proteasome | 98 | ND |
| ko03060 | Protein export | 65 | ND |
| ko03070 | Bacterial secretion system | 18 | ND |
| ko03410 | Base excision repair | 53 | ND |
| ko03420 | Nucleotide excision repair | 104 | 2 |
| ko03430 | Mismatch repair | 74 | ND |
| ko03440 | Homologous recombination | 77 | 2 |
| ko03450 | Non-homologous end-joining | 11 | ND |
| ko03460 | Fanconi anemia pathway | 84 | 4 |
| ko04010 | MAPK signaling pathway | 66 | 8 |
| ko04011 | MAPK signaling pathway - yeast | 66 | 5 |
| ko04012 | ErbB signaling pathway | 6 | ND |
| ko04013 | MAPK signaling pathway - fly | 67 | 2 |
| ko04014 | Ras signaling pathway | 68 | 3 |
| ko04015 | Rap1 signaling pathway | 26 | 1 |
| ko04022 | cGMP-PKG signaling pathway | 57 | 3 |
| ko04024 | cAMP signaling pathway | 65 | 2 |
| ko04064 | NF-kappa B signaling pathway | 35 | 5 |
| ko04066 | HIF-1 signaling pathway | 65 | ND |
| ko04068 | FoxO signaling pathway | 100 | ND |
| ko04070 | Phosphatidylinositol signaling system | 99 | 7 |
| ko04071 | Sphingolipid signaling pathway | 98 | 2 |
| ko04072 | Phospholipase D signaling pathway | 55 | 5 |
| ko04080 | Neuroactive ligand-receptor interaction | 2 | ND |
| ko04110 | Cell cycle | 202 | 3 |
| ko04111 | Cell cycle - yeast | 165 | 3 |
| ko04112 | Cell cycle - Caulobacter | 30 | ND |
| ko04113 | Meiosis - yeast | 125 | 3 |
| ko04114 | Oocyte meiosis | 146 | 4 |
| ko04115 | p53 signaling pathway | 65 | 3 |
| ko04120 | Ubiquitin mediated proteolysis | 212 | 6 |
| ko04122 | Sulfur relay system | 20 | 1 |
| ko04130 | SNARE interactions in vesicular transport | 68 | 6 |
| ko04139 | Regulation of mitophagy - yeast | 71 | 2 |
| ko04140 | Regulation of autophagy | 43 | 1 |
| ko04141 | Protein processing in endoplasmic reticulum | 334 | 14 |
| ko04142 | Lysosome | 103 | 1 |
| ko04144 | Endocytosis | 263 | 22 |
| ko04145 | Phagosome | 136 | 3 |
| ko04146 | Peroxisome | 143 | 4 |
| ko04150 | mTOR signaling pathway | 72 | ND |
| ko04151 | PI3K-Akt signaling pathway | 119 | 2 |
| ko04152 | AMPK signaling pathway | 130 | 6 |
| ko04210 | Apoptosis | 45 | 1 |
| ko04214 | Apoptosis - fly | 57 | 1 |
| ko04310 | Wnt signaling pathway | 117 | 2 |
| ko04330 | Notch signaling pathway | 22 | ND |
| ko04340 | Hedgehog signaling pathway | 40 | ND |
| ko04341 | Hedgehog signaling pathway - fly | 56 | ND |
| ko04350 | TGF-beta signaling pathway | 53 | ND |
| ko04370 | VEGF signaling pathway | 28 | 1 |
| ko04390 | Hippo signaling pathway | 54 | ND |
| ko04391 | Hippo signaling pathway - fly | 54 | ND |
| ko04392 | Hippo signaling pathway -multiple species | 15 | 1 |
| ko04510 | Focal adhesion | 33 | ND |
| ko04520 | Adherens junction | 29 | ND |
| ko04530 | Tight junction | 49 | ND |
| ko04540 | Gap junction | 30 | 2 |
| ko04550 | Signaling pathways regulating pluripotency of stem cells | 7 | ND |
| ko04630 | Jak-STAT signaling pathway | 10 | ND |
| ko04810 | Regulation of actin cytoskeleton | 90 | 2 |

ND means not detected.
